# Female pelvic cancer survivors’ experiences of pelvic floor muscle training after pelvic radiotherapy

**DOI:** 10.1007/s00520-024-09041-w

**Published:** 2024-12-02

**Authors:** A. Lindgren, S. Börjeson, G. Dunberger

**Affiliations:** 1 Department of Health, Medicine, and Caring Sciences, Division of Prevention, Rehabilitation and Community Medicine, Linköping University, 58183 Linköping, Sweden; 2 Department of Health, Medicine, and Caring Sciences, Division of Caring Sciences and Reproductive Health, Linköping University, Linköping, Sweden; 3 Department of Health Care Sciences, Marie Cederschiöld University, Stockholm, Sweden

**Keywords:** Incontinence, Pelvic cancer, Pelvic floor muscle training, Physiotherapeutic support, Radiotherapy

## Abstract

**Purpose:**

To describe experiences of a 3-month pelvic floor muscle training (PFMT) period, with or without support from a physiotherapist, among females with urinary and/or fecal incontinence after pelvic radiotherapy.

**Method:**

This qualitative interview study included eleven women (aged 47–83 years) with urinary and/or fecal incontinence after radiotherapy treatment for pelvic cancer (radiotherapy completed 3–60 months ago). The eleven informants were part of a larger randomized controlled intervention study where they practiced PFMT, with or without support from a physiotherapist, for 3 months. The support from a physiotherapist included individual support with biofeedback as well as group training. The women were interviewed individually soon after the completion of the pelvic floor muscle training period, and data were analyzed with qualitative content analysis.

**Result:**

A structured training program, individual support from a physiotherapist, and strategies to establish a daily workout routine were described as valuable when practicing pelvic floor muscle training. Participating in the study gave a sense of meaningfulness and motivation to practice, partly due to the knowledge of a follow-up after the study period. Group and home training were described as both a facilitator and a barrier to PFMT. The women experienced that PFMT had influenced pelvic floor function in terms of increased pelvic floor strength, reduced urinary and fecal incontinence, and an increased ability to hold urine and feces during urgency. PFMT had a relieving effect on pelvic floor pain, although it also contributed to some increase in pain. The perceived improvement in pelvic muscle function led to decreased anxiety, increased safety, feelings of greater freedom in everyday life, a changed attitude toward physical activity, and improved sexual health. All women expressed an intention to continue practicing PFMT and a desire for information and opportunities for PFMT, under the guidance of a physiotherapist, to be implemented in conventional pelvic cancer rehabilitation and made available to all women after pelvic cancer treatment.

**Conclusion:**

The women who live with the experience of pelvic cancer experienced PFMT as a meaningful intervention for managing urinary and/or fecal incontinence after pelvic radiotherapy. They considered that information and support from a physiotherapist are essential in pelvic cancer rehabilitation, such as PFMT, and should be offered to all women after pelvic cancer treatment.

## Introduction

Urinary and fecal leakage is common after pelvic cancer treatment, especially after pelvic radiotherapy [1–5]. These symptoms of pelvic floor dysfunction are known to have an adverse impact on the quality of life, social functioning, and everyday life [1, 2, 6–8] as well as physical activity levels [7, 9] among women who live with the experience of pelvic cancer . Indeed, in a previous qualitative study, we interviewed thirteen females who identified incontinence as a barrier to practicing physical activity after pelvic cancer treatment, especially if those activities were not conducted close to a bathroom. In this study, the females indicated little or no experience with rehabilitative measures to reduce incontinence, such as pelvic floor muscle training (PFMT) [10]. In the clinical practice guidelines for individuals treated for prostate cancer, there is an organized focus on support and education, including clinicians offering patients PFMT before and after surgery, to help patients with urinary incontinence maintain quality of life and increase physical activity [11, 12]. However, for females treated for pelvic cancer, such an organized focus on support and education is often lacking. More than 30,000 living women in Sweden have undergone treatment for gynecological cancer, indicating that supportive and rehabilitation interventions ought to be prioritized within the healthcare system [13]. In a study of 578 females who had undergone treatment for pelvic cancer, we found that 33% practiced physical activity less than once a week. When experiencing fecal incontinence, they were even less likely to practice physical activity [9]. Thus, similar rehabilitation strategies as those provided for men after prostate cancer treatment are needed for females after pelvic cancer treatment to help them reduce incontinence and maintain quality of life.

Generally, PFMT is recommended as an effective first-line treatment for urinary and fecal incontinence [14, 15]. When effective, it may indeed increase ability and improve QoL [14]. However, research on the effect of PFMT on females after pelvic cancer treatment is sparse [16], especially after pelvic radiotherapy. Bernard et al. (2021) report, in a single-subject experimental study (*n* = 11), that the severity of urinary incontinence can be improved, among women who have undergone radiotherapy, by an in-home rehabilitation program including PFMT. The authors further emphasize that support from the treating physiotherapist was important. This support was essential for overcoming difficulties and in giving instructions to continue the exercise program [17].

According to Brennen et al. (2022), further studies exploring the feasibility of multidisciplinary therapy, including physiotherapy interventions, for pelvic floor dysfunction after gynecological cancer treatment are warranted [18]. To achieve compliance and ensure that support is provided in PFMT, it is likely that physiotherapeutic guidance and support are needed. Women’s experiences of having practiced PFMT after pelvic cancer treatment could give an indication of whether PFMT is a meaningful form of rehabilitation also after radiotherapy treatment. It may also help clarify whether guidance is justifiable to encourage women in routine healthcare to practice PFMT after pelvic cancer treatment. Exploring the women’s experiences might also contribute to new insights for future development of how to design, even more, meaningful PFMT interventions for females treated for pelvic cancer.

The purpose of this study is to explore and describe experiences of a 3-month pelvic floor muscle training (PFMT) period, with or without support from a physiotherapist among females treated for pelvic cancer with urinary and/or fecal incontinence after pelvic radiotherapy.

## Participants and methods

### Design

The topic of the present study is, to our knowledge, unexplored. An exploratory qualitative descriptive interview study was conducted to generate a deeper understanding of women’s experiences of practicing PFMT, with or without support from a physiotherapist, after pelvic radiotherapy. Data was collected through individual face-to-face interviews, using a semi-structured interview guide (Appendix 1), and were analyzed with content analysis inspired by Krippendorff [19] (see Table 2).

### Participants

The participants consisted of a convenience sample of eleven women who were already participating in a randomized controlled intervention study, where they had practiced pelvic floor muscle training, with or without support from a physiotherapist, for 3 months. The support from a physiotherapist included individual support with biofeedback as well as group training (for intervention description, see Appendix 2).

Participants were enrolled by asking if they were also willing to participate in a qualitative interview study when they were asked to participate in the RCT. Inclusion criteria were women over 18 years of age who had completed curative radiation therapy for pelvic cancer 3–60 months ago, who reported urine or fecal leakage, and who accepted participation in the randomized study with the associated qualitative study.

None of the women declined to participate in the subsequent qualitative interview study. Written consent was obtained from the study participants before the interviews were conducted. The women were interviewed within a week after having completed their PFMT period. Inclusion stopped when the last two interviews did not provide any new relevant information from new patients. The content from the interviews was then assessed as rich (*n* = 11 study participants: R1–R11).

Demographic and treatment-related information had already been collected in the RCT, before the participants started their training period, through a written questionnaire. Our methods for the development of study-specific questionnaires for collecting background data, such as remaining symptoms after cancer treatment, have been documented in more than 80 scientific papers [20].

### Ethical considerations

Participants were informed both in writing (in the information letter) and orally (before the interviews began) that their participation was voluntary and that they could at any time, during the interview, cancel their participation or choose not to answer certain questions, without stating any reason. They were also informed in writing, before the interview, that the interviews would be recorded and that the data would be stored so that no unauthorized person outside the research group could access it. The study was performed according to the Helsinki Declaration of Ethical Principles for Medical Research Involving Human Subjects [21] and was approved by the Regional Ethical Review Board in Gothenburg (D 990–17).

### Data collection

Interviews were conducted by a physiotherapist with more than 10 years’ experience working at a women’s clinic, through individual face-to-face (physical) audio-recorded interviews, at the clinic, in connection with the final measurements being made in the RCT. The interviewer was continuously supervised during the interview process by the research group who had experience in conducting several qualitative studies. However, only the respondent and the interviewer were present during the interview. The interviewing physiotherapist was employed in the ongoing RCT study only for conducting baseline and final measurements and to conduct the interviews. The interviewer had met the women once before, when conducting the baseline measurements in the RCT, before the women started to practice PFMT. The respondents were aware of the physiotherapist’s extensive experience working clinically in women’s health. For the rest of her working time, while the study was conducted, the interviewer worked clinically as a physiotherapist at a women’s clinic and had no further contact with the study participants.

The interviewer used a semi-structured interview guide with open-ended questions formulated by the authors based on the study purpose (presented as Appendix 1). The development of the interview guide was based on four methodological steps recommended by Kallio et al. “(1) identifying the prerequisites for using semi-structured interviews; (2) retrieving and using previous knowledge; (3) formulating the preliminary semi-structured interview guide; (4) pilot testing the guide; and (5) presenting the complete semi-structured interview guide” [22]. A semi-structured interview guide was used because we wanted to explore and describe women’s experiences. Previous knowledge was retrieved from former qualitative research in the research group where women described, for example, little or no experience of pelvic floor muscle training but a positive attitude toward trying it [10] and knowledge from years of work in women’s health and in cancer rehabilitation within the research group.

The interview guide started with an initial broader question: “How has it been for you to exercise pelvic floor muscle training now during the study period?” and continued with more specific questions like “How have you been able to exercise according to the current training program?” and “What has been good and what has been less good about exercising the way you have done?” The interviewer evaluated the interview guide in two pilot interviews. The pilot interviews were conducted with two women with incontinence after pelvic radiotherapy who had practiced PFMT as pilot study participants in the RCT. Since they had completed radiotherapy treatment more than 60 months ago, they did not fulfill the inclusion criteria, and the pilot interviews were not included in the results but were valuable for evaluating the usability of the interview guide. The interview guide was found to be applicable since the respondents in the pilot interviews perceived the questions as easy to understand and clear. No revision was made after the pilot interviews. The interviews in the present study lasted for 20 to 45 min (median 30 min).

### Data analysis

Data were analyzed inductively because knowledge of experiences of PFMT, with or without support from a physiotherapist, among women who live with the experience of pelvic cancer was limited. First, the interviews were transcribed verbatim (AL), and then, the data were analyzed by two of the authors (AL and GD) using qualitative content analysis influenced by Krippendorff [19] (see Table 1). The text was then read several times, and sentences and phrases, i.e., textual units relevant to the purpose of the study, were identified to create a sense of the whole (AL). Choosing units that were too long or too short was avoided. This was because long units might contain more than one phenomenon, and short units might make the material fragmented [19]. The textual units were condensed to shorten the text with the intention of still maintaining the content of the units (AL). After that, the condensed textual units were coded (AL). The authors (AL and GD) grouped the textual units into distinct categories independently of each other. The categories were compared and discussed until a consensus was reached. The categories then reflected the central message of the interviews in relation to the purpose of the study. The condensed textual units were considered to belong to a relevant category, and the units were exclusive in relation to each other. To strengthen the trustworthiness of the analysis and to ensure the dependability of the results, the second author (SB) read the analysis and confirmed that it reflected the content of the transcripts. As none of the last three interviewees contributed to any new codes, categories, or subcategories, saturation in the material was considered to have been achieved [23].

**Table 1:** Content analysis in five steps

	Example
1. Interviews were transcribed verbatim or listened to by all authors. Transcripts were read several times to *create a sense of the whole*	Excerpt from note: *Then I have tried to establish a routine when it comes to this training at home by myself*
2. Phrases and sentences with information relevant to the purpose of the study, so-called *textual units*, were picked out by the first author. The intent was to select units that were not too long, as they might contain more than one phenomenon, or too short due to the risk that the material may become fragmented	*Try to establish a training routine at home by myself*
3. The textual units were *condensed* by the first author to compress the text without losing its content. The first and the last authors, independently of each other, placed the condensed textual units into groups representing different subcategories	*Establish a home training routine*
4*. Subcategories* were discussed, by the first and the last authors until a consensus was reached that the categories reflected the central message of the interviews in light of the purpose of the study	*Impact and challenge of home training*
5. The subcategories were structured into *main categories* by the first and last authors	*Facilitators and barriers to practicing pelvic floor muscle training*

## Results

The median age was 69 years (range 47–83 years). Seven of the eleven respondents had, according to the randomization, practiced PFMT with the support of a physiotherapist. Further demographic data, relevant to the qualitative interview study, are presented in Table 2.

**Table 2:** Characteristics of the participants (*n* = 11)

Randomized to support from physiotherapist	
Yes	7
No	4
Median age (range)	69 (47–83)
Pelvic cancer treatment	
Radiotherapy	1
Radiotherapy and hormonal therapy	1
Radiotherapy and chemotherapy	1
Radiotherapy, brachytherapy and chemotherapy	3
Radiotherapy, brachytherapy, chemotherapy and hormonal therapy	1
Surgery and radiotherapy	1
Surgery, radiotherapy and chemotherapy	2
Surgery, brachytherapy and chemotherapy	1
Type of cancer	
Rectal cancer	4
Colon cancer	3
Cervical cancer	3
Other pelvic cancer (not specified)	1
Type incontinence	
Only urinary	5
Only fecal	0
Urinary and fecal	6
Civil status	
Married/ living with partner	6
Partner but lives alone	3
Lives alone, no partner	2
Work situation	
Working	2
Retired	8
On sick leave	1
Education	
Elementary school	2
High school	6
University/college	3

The analysis resulted in three categories: *Facilitators and barriers to practicing pelvic floor muscle training*, *Experienced health-related impact of pelvic floor muscle training*, and *Future considerations in relation to pelvic floor training* with seven subcategories (Table 3). *Facilitators and barriers to practicing pelvic floor muscle training* describe the experienced impact of support from a structured training program in a study context, the importance of support from a physiotherapist, and the impact of training in group and home training, including challenges. *Experienced health-related impact of pelvic floor muscle training* describes experienced physical impact on pelvic floor muscle function, psychological and behavioral impact, and impact on sexual health after performing pelvic floor muscle training. *Future considerations in relation to pelvic floor training* describe respondents’ intentions regarding future PFMT practice and future desires for pelvic cancer rehabilitation.

**Table 3:** Categories and subcategories describing PCS’ experiences of practicing PFMT with or without physiotherapy support

Category	Subcategory
Facilitators and barriers to practicing pelvic floor muscle training	*- Support from a structured training program in a study context* *- Importance of support from a physiotherapist* *- Impact of training in a group* *- Impact and challenge of home training*
Experienced health-related impact of pelvic floor training	*- Physical impact on pelvic floor muscle function* *- Psychological and behavioral impact* *- Impact on sexual health*
Future considerations in relation to pelvic floor training	Not applicable*

^*^The main category had no subcategories

### Category 1: facilitators and barriers to practicing pelvic floor muscle training

#### Support from a structured training program in a study context

Participating women experienced that practicing according to a structured training program was valuable in giving them guidance regarding frequency, dose, and progression. The structured training program made them aware of the importance of practicing both strength and endurance and made it easier to remember the exercises. Some women found the program easy to follow, while others, practicing PFMT without support from a physiotherapist, found it hard to know when to increase the severity of the exercise.

How to adjust the contractions, not just to squeeze and relax. Sometimes it was supposed to go fast, sometimes you were supposed to hold the contraction for a long time. It felt very good. (R8, S = supervised)

It's good to have a program to stick to. Otherwise, it's easy to forget the exercises even if you're supposed to practice every day. (R11, S)

Most women found the period of 3 months reasonable and found it easier to commit to a regimen when they knew it was for a certain period. They also experienced that the time they had to spend on PFMT, each day, was doable, without problems.

It is good that you have a schedule, for a reasonably long period, then it is easier for me to get started with things if I know that it is now these weeks. (R2, S)

The time was just long enough to be able to carry it out every day. (R2, S)

Participating in a study gave them motivation to practice, due to the knowledge of a follow-up after the study period. The women also expressed feelings of commitment to fulfill their study participation to the best of their ability. Feelings of meaningfulness, as the results could benefit other cancer survivors in the future, also contributed to a higher motivation to practice PFMT.

#### Importance of support from a physiotherapist

When practicing PFMT with the support of a physiotherapist, the women felt that individual biofeedback sessions were an essential element. It was valuable to get guidance on how to contract the pelvic floor muscles without contracting surrounding muscles. It was also more fun and motivating to continue exercising if they knew they were doing it correctly.

Then I realized, I got more feeling for what it should feel like, when I could see the curve and got feedback directly (refers to biofeedback). I thought it was a very good way to find the right muscles. (R5, S)

You know your pelvic floor a little better know, you are more aware of what you do. Now when I contract the muscles, I think about how the contraction feels and if I contract other muscles than just the pelvic floor muscles. (R6, S)

It was a lot of fun. When you see that you're contracting the muscles right, via biofeedback, you become even more motivated to continue. (R2, S)

The women described that they became even more motivated and pushed to progress with the difficulty of the PFMT regarding frequency, intensity, and duration, when having support from a physiotherapist.

I don't think I would have succeeded so well if I hadn't had this push from a physiotherapist every week. If I had only trained at home myself, then I would have cheated more to be honest. (R4, S)

Being able to ask questions to the physiotherapist regarding training techniques and progression and receive immediate feedback was described as valuable. It was also positive to get tips and advice on how to integrate training into everyday life, like how to use their pelvic floor muscles when lifting heavy objects. It was easier to understand and absorb when the written information was given in combination with further oral explanation by the physiotherapist.

The advice, for example, that you do not always have to lie down and practice PFMT, you can do it in standing position as well, for example when brushing your teeth or cooking. You get into that way of thinking, that now I can take the opportunity for some PFMT practice. (R8, S)

Being in private sessions with the physiotherapist was even more valuable. It was easier to talk about and ask questions regarding PFMT and incontinence in privacy, along with the physiotherapist.

The times I've been alone with the physiotherapist, those times I've gotten more out of. You might ask questions in a different way when you're alone, than if there are more people you don't know, since it’s so personal. (R S)

The women practicing PFMT without support from a physiotherapist experienced that it was sometimes challenging to follow the training program when not given any further oral training information or practical training instructions beyond the written training program.

If I contracted the muscle properly, I do not know. But I have tried to do what it says on the paper, but you do not know. (R10, U = unsupervised)

#### Impact of training in a group

The group training sessions, for some, ensured practicing PFMT on a regular basis, even if they had to travel far. However, others described the group training as problematic due to traveling and the need to adapt to scheduled training sessions. They expressed a desire for more workout sessions, to choose from. Still, others thought it would have been easier to attend all sessions if they were scheduled once a week or once every second week.

I see no disadvantages (with the group training) except that I had to travel far. But it was worth it. (R4, S)

It has been good that you had to get started and really do it (practice PMT). You know, after all, that you must start. But just that one would also go here, so we had to do it (practice PFMT). I thought it was good. (R5, S)

There were different opinions on whether practicing PFMT in a group, together with other pelvic cancer survivors, had contributed to their training experience in a positive way or not. Some described that it was too private to talk in front of strangers about incontinence problems and therefore difficult to see the benefits of group exercise with other pelvic cancer survivors. For others, however, it was a relief to discover that they were not alone, suffering from incontinence after pelvic cancer treatment. The group training was described as positive, providing a social activity to attend with others working toward the same goal, which was to reduce incontinence.

I do not really know if it had any meaning, the fact that we were training in a group. You are quite anonymous although you are in a group. (R 6, S)

It is always good to know that you are not alone in these concerns. You do feel a bit lonely sometimes. (R11, S)

It was nice when you got there it was someone you recognize. And practiced with the same goal. (R8, S)

#### Impact and challenge of home training

Being able to decide when to train made it easier to practice PFMT regularly. It was described as difficult and boring to practice PFMT by yourself at home, but also a convenient way to practice.

Then I could get a routine in peace and quiet for myself. (R10, U)

Those who practiced PFMT, by themselves at home, without support from a physiotherapist, however, described that it was difficult to know whether they were contracting the pelvic floor muscle correctly.

I don't know if I did the right thing, but I think so. (R3, U)

Integrating PFMT into daily activities was described as an effective way to establish a daily routine. It was easier to remember if done while doing other things, such as brushing their teeth or driving to work. Incorporating the exercises into everyday activities was also described as facilitating regular training, as a lot of extra time did not have to be devoted to PFMT daily. The women described that it eventually became a habit to practice PFMT but that it took a couple of weeks for the habit to be established.

### Category 2: experienced health-related impact of pelvic floor muscle training

#### Physical impact on pelvic floor muscle function

The women described increased pelvic floor muscle strength, noting that it was easier to contract the pelvic floor muscles and easier to consciously contract their pelvic floor muscles in everyday life when necessary.

It's been going well. And I've felt it has given me more strength. (R2, S)

They described an impact on urinary incontinence such as less frequent urinary leakage, a lesser amount of urinary leakage, and a better ability to hold urine in case of urgency.

I think it has gotten better, less leakage, or above all, more rarely, no longer daily. So, I have noticed a clear improvement (refers to the improvement of urinary leakage). (R5, S)

Women, practicing PFMT with support from a physiotherapist, further described an impact on fecal incontinence levels in terms of less frequent events of leakage and better ability to hold feces in case of urgency.

During this time, I think I may have leaked little less feces. (R6, S)

Meanwhile, a woman practicing PFMT without support from a physiotherapist described that she did not experience any impact from PFMT.

I have not noticed any improvement with my problems. (R1, U)

Another woman, who was offered support from a physiotherapist but did not attend any of the group sessions described that she did not experience any impact from PFMT either.

It hasn't gotten any better. (R9, S)

Another woman described that she did not experience any impact from PFMT in terms of decreased incontinence but did experience slightly increased pelvic pain in the area around the tailbone, which was described as a problem even before entering the study. On the other hand, another woman described both a better ability to hold urine in case of urgency and due to PFMT; she no longer experienced any pain during intercourse.

#### Psychological and behavioral impact

When experiencing decreased incontinence levels, they also experienced less concern about embarrassment due to involuntary leakage and less concern that incontinence problems would worsen over time.

If you leak, it gives you an uncertainty and fear that you smell. I thought that was very difficult. Since I leak less often it has decreased a little (the uncertainty and fear of smelling) in the interaction with other people. (R5, S)

It was also described as contributing to increased security and a sense of freedom in everyday life, not having to constantly know where the nearest toilet is located.

You can relax more and know that I can stay, if I am out, for several hours and not have to think that I should go home or need to go to the bathroom. You feel freer. (R2, S)

Women also described that the altered pelvic floor function had affected their attitude toward physical activity. After being part of the study one, woman described that she perceived that PFMT led to increased pelvic pain, which sometimes made her avoid being physically active.

If I had a lot of pain, then maybe I did not go for that walk as I first intended to. But that has been the case for many years now. (R6, S)

Meanwhile, as they began to experience less incontinence after having practiced PFMT, some women dared to resume physical activities that they had stopped doing because of incontinence. One woman described that when experiencing less incontinence, she had started jogging and dared to try the trampoline and discovered that she no longer experienced the same weight sensation in the pelvic floor when jumping.

I jog a little, that I started with, and there I felt that you do not feel the same pressure downwards, that you feel stronger in the pelvic floor. And when I jumped trampoline with the kids, I also felt a huge difference that you do not get this pressure down. I get a little but not as much as I had before. (R2, S)

#### Impact on sexual health

Women described difficulties being sexually active after pelvic cancer treatment due to incontinence and pain during intercourse. Improved pelvic floor function, after having practiced PFMT, was described as a contributing factor to improved sexual health. One woman described that since she no longer experienced pain during intercourse, it was more pleasurable and tempting to have sex after having practiced PFMT. Another woman described it as easier to achieve orgasm and that her partner also found intercourse more pleasurable thanks to stronger pelvic floor muscles.

The intercourse has improved. Before (before she had practiced PFMT) I often had pain afterwards, but it has disappeared. Why I do not know, but I must link it to this (to PFMT). I do not know if there is any muscle in there, I do not know, but it has become much better, it has disappeared. (R7, U)

I get orgasm much easier… My partner thinks it feels much better for him too than it did before. (R4, S)

### Category 3: future considerations in relation to pelvic floor training

All women, even the ones not experiencing any effects of the PFMT, described an intent to continue practicing PFMT by trying to integrate it into everyday activities.

I will continue, I guess, as I did the last few weeks, put it into everyday life, when I brush my teeth, then maybe not like now I should do eight and then six (pelvic floor muscle contractions). I will continue to practice PFMT, but maybe not after a certain schedule. (R6, S)

Several women pointed out the importance of PFMT with the support of a physiotherapist and wished it could be a mandatory part of conventional pelvic cancer rehabilitation.

It should be an obvious part of everything, you have this clinic for pelvic cancer therapy-induced consequences, it (to get to practice PFMT with the support of a physiotherapist) must be included as a part there. It cannot be that they sit there and say: it is just that you go home and practice PFMT. For someone it might work, but for people like me it does not. I need help with how to do; to find out how to physically contract my muscles and know where to start and in what way and for how long and how often and in what position, the whole package. (R4, S)

The women also described a demand for more information about incontinence and rehabilitation, such as PFMT, for treatment-related urinary and fecal incontinence, in regular pelvic cancer care.

Information about how it can be after treatment so that you are aware of it. Sure, it is mentioned in passing, but I lack more details. (R4, S)

One woman even described that it would have been valuable to learn how to practice PFMT already before cancer treatment to learn how a correct pelvic floor muscle contraction feels, before any potential injury is induced to the pelvic floor muscle.

## Discussion

This study contributes to new knowledge regarding women’s experiences of PFMT, with or without support from a physiotherapist, when experiencing urinary and/or fecal incontinence after pelvic radiotherapy. Furthermore, it contributes to increased knowledge about how women believe that physiotherapists might contribute, in a valuable way, to incontinence rehabilitation after pelvic cancer treatment. Women practicing PFMT after pelvic cancer treatment had mainly positive experiences with a structured training program, individual support from a physiotherapist, and establishing a daily routine for the training. Group and home training were both a facilitator and a barrier to training compliance. Traveling to group training and having to adapt to scheduled training sessions were however described as barriers to PFMT. Being in a study group and following a training program without any given oral instructions or feedback from a physiotherapist were also perceived as barriers to PFMT. Meanwhile, home training was described as a convenient way to practice. The women described that the training increased pelvic floor strength, decreased urinary and fecal incontinence, and gave them a better ability to hold urine and feces in case of urgency. The training both increased and decreased pelvic floor pain. The perceived improved pelvic floor muscle function led to feelings of less concern about incontinence, increased security, and a sense of greater freedom in everyday life including physical activity and better sexual health. The women had intentions to continue with PFMT and considered that information and training support should be offered to all women who live with the experience of pelvic cancer.

As a negative side effect of radiotherapy, the tissue in the pelvic area will potentially become fibrotic and rigid [24, 25]. The assumption that PFMT could strengthen fibrotic muscles could be seen as a mission impossible. Small pilot studies on the effect of PFMT among women after pelvic cancer treatment indicate that PFMT could influence pelvic floor muscle strength and incontinence, when these are experienced as a side effect after cancer treatment, but not specifically only after radiotherapy [16, 26, 27].

One single-subject experimental study (*n* = 11) reported that urinary incontinence can be improved among women after radiotherapy by a rehabilitation program including PFMT and that the support from the treating physiotherapist was of great importance [17], which is in line with our qualitative results. When practicing PFMT, the women found support from a physiotherapist and biofeedback valuable, a result that is in line with previous research. Women without the experience of pelvic cancer also have difficulty in effectively activating the pelvic floor muscles without supervision [14]. However, women who have gone through pelvic radiotherapy with potential fibrotization of the pelvic floor muscles [24, 28] may find this even more difficult. Hence, support for women is particularly important after pelvic radiotherapy.

Our results show that women experience PFMT as a meaningful intervention and find the support from a physiotherapist valuable when experiencing both urinary and/or fecal incontinence after pelvic cancer treatment. This is a valuable new insight that could help in the future development of how to design meaningful PFMT interventions for women who live with the experience of pelvic cancer.

We also found that the participants in this study thought that information and training support should be offered to all women after pelvic cancer treatment. This further supports our opinion that there is a need for organized support from a physiotherapist among women suffering from incontinence after pelvic cancer treatment; this would be in line with clinical practice guidelines for males after pelvic cancer treatment. For individuals after prostate cancer treatment, there is an organized focus on support and education to help patients with urinary incontinence maintain QoL and increase physical activity levels [11, 12]. A review has concluded that comprehensive and specific recommendations for training programs after prostate cancer treatment may need to be a lifelong exercise habit [29]. Since late side effects after radiotherapy are irreversible and often progressive [25, 28, 30], there is no reason to believe that the case would be different for women after pelvic cancer treatment.

To ensure that no important aspects were overlooked in reporting important aspects of the research team, study methods, context of the study, findings, analysis, and interpretations, COREQ, a 32-item checklist, was used as a guideline [31]. One strength of the present study is that the content was rich and reflects the respondents’ experience of PFMT. The trustworthiness of our analysis was secured by triangulation [19] (two analyzers and validated by a third). Given that this is a qualitative study exploring women’s experiences of PFMT within the context of a specific RCT, the results cannot be generalized to all women following pelvic cancer treatment. Nevertheless, due to the wide range of demographic data among the participants and the fact that we achieved redundancy in the data collection, the findings may be transferable to women with similar incontinence experiences post-pelvic radiotherapy, in comparable contexts.

The researchers have experience from different perspectives, which is also considered a strength. One of the researchers is an oncology specialist nurse, and two of the researchers have extensive experience in oncology nursing/clinical cancer rehabilitation and qualitative and quantitative research in the field of cancer rehabilitation. The third researcher, on the other hand, is a physiotherapist with broad experience in clinical rehabilitation in women’s health, in the field of incontinence. The overall knowledge of the research team, through broader thinking, has contributed to both relevant questions for the interview guide as well as relevant instructions for the interviewer regarding how to ask follow-up questions to achieve rich interviews. It is important to make sure that a researcher’s pre-understanding does not lead to pre-conceived notions of what is meaningful to the patient and therefore exclude questions significant to the patient. The researchers, with their different pre-understandings, complemented each other when choosing questions for the interview guide that would capture the most meaningful aspects of the phenomenon meaningful for the respondents. The fact that the interviewer had limited experience in conducting qualitative interviews, using a semi-structured interview guide, could have resulted in insufficient interview content. It could also have been a disadvantage that the interviewer was a physiotherapist, with more than 10 years’ experience in women’s health with a pre-understanding that might have led the interview in the wrong direction. With comprehensive experience, there is always a risk that certain questions are not asked because the interviewer already believes they know the answer. Yet many people experience incontinence as shameful and sensitive to talk about. The interviewer’s experience hopefully contributed to a sense of security so that the women dared to describe their experiences of incontinence and PFMT in depth.

## Conclusion

Women who live with the experience of pelvic cancer found PFMT to be a valuable rehabilitative tool for relieving both urinary and fecal incontinence. They experienced that physiotherapeutic support and guidance were important when practicing PFMT to be able to contract the pelvic floor muscles correctly and to receive individual guidance regarding dose, frequency, and progression. Furthermore, they consider that information about and support from a physiotherapist in combination with pelvic floor muscle training should be offered to all women after pelvic cancer treatment.

## Data Availability

No datasets were generated or analysed during the current study.

## Appendix 1

### Interview guide

Preliminary overall question:

“How has it been for you to practice pelvic floor muscle ) training (PFMT) now during the study period?”

Further questions:

1. How have you been able to practice according to the current training plan?
2. What has been good and what has been less good about exercising the way you have done?
3. What effect do you think the PFMT has had?
4. In what way have the effects of the PFMT affected your everyday life?
5. How do you think about PFMT training in the future?

The following are suggestions for follow-up questions to get the respondent to talk more:

Can you expand on this a bit more?

In what way?

What do/did you feel?

What do you mean?

The interviewer finally summarizes all the questions and asks the respondent if she wants to add anything.

## Appendix 2

### Description of the interventions in the RCT that the respondents had been a part of before enrolling in the present qualitative interview study

The participants in the RCT were randomized either to PFMT with the support of a physiotherapist or PFMT without the support of a physical therapist:

#### PFMT with the support of a physiotherapist

Twelve-week PFMT twice a week under the guidance of a physiotherapist, in addition to home training instructions (see PFMT in the form of home training below) conducted the other days of the week. During the first 2 weeks, the women receive individual PFMT sessions of 30–40 min twice a week to teach proper PFMT techniques with the assistance of a physiotherapist and with biofeedback. From the third week, the women were offered to participate in group training under the guidance of a physiotherapist, about 30 min twice a week for 10 weeks. The PFMT program is based on the scientific evidence available on PFMT techniques: practical exercises focused on strength, endurance of the pelvic floor muscles, and speed /functional training, such as contraction during, for example, sitting up, knees bent, fast walking/jogging, and jumping. The women exercise individually but together with others based on their individual level.

#### PFMT without the support of a physical therapist

The participants obtained written home training instructions on PFMT for 12 weeks: strength, endurance, and speed/functional training to be performed individually each day. Exercises, dose, and progression in the home exercise program were the same as in the intervention group. What separated the control group from the intervention group was that they did not receive support and guidance from a physiotherapist.
